# Emerging incidence trends of eosinophilic esophagitis over 25 years: Results of a nationwide register‐based pathology cohort

**DOI:** 10.1111/nmo.14072

**Published:** 2021-01-10

**Authors:** Willemijn E. de Rooij, Marielle E. Barendsen, Marijn J. Warners, Bram D. van Rhijn, Joanne Verheij, Annette H. Bruggink, Albert J. Bredenoord

**Affiliations:** ^1^ Department of Gastroenterology and Hepatology Amsterdam University Medical Center Amsterdam The Netherlands; ^2^ Department of Gastroenterology and Hepatology University Medical Center Utrecht and st. Antonius Hospital Nieuwegein Nieuwegein The Netherlands; ^3^ Department of Dermatology and Allergology University Medical Center Utrecht Utrecht The Netherlands; ^4^ Department of Pathology Amsterdam University Medical Center Amsterdam The Netherlands; ^5^ The nationwide Network and Registry of Histo‐ and Cytopathology in the Netherlands (PALGA) Houten The Netherlands

**Keywords:** eosinophilic esophagitis, epidemiology, esophageal eosinophilia and allergy, incidence

## Abstract

**Rationale:**

Eosinophilic esophagitis (EoE) has emerged from a case‐reportable illness in the early 1990s to a distinct clinicopathological entity. Increasing worldwide incidences have been observed, although due to various study designs estimates are inconsistent.

**Aim:**

To determine population‐based annual incidence rates over a time period of 25 years.

**Methods:**

A nationwide register‐based pathology (PALGA) search was performed to identify reports describing esophageal eosinophilia between 1995 and 2019. EoE was identified if the diagnosis was confirmed by the pathologist. Crude incidence rates were estimated by the number of new EoE cases per year and matched with population data.

**Results:**

Between 1995 and 2019, 7361 unique patients’ reports mentioned esophageal eosinophilia, of these 4061 were classified as EoE (71% male, mean age 37.9 ± 18.4 years). In total, 639 (16%) children (<18 years) were diagnosed. The EoE incidence increased from 0.01 in 1995 (95% CI: 0.0 – 0.04) to 3.16 (95% CI: 2.90 – 3.44) per 100.000 inhabitants in 2019. EoE was significantly more prevalent in males (OR 2.48 | 95% CI: 2.32 – 2.65; vs. females *p* < 0.001) and adults (OR 1.42 | 95% CI: 1.31 – 1.55; vs. children *p* < 0.001). Highest incidences were observed in 2019, being 4.37 (95% CI: 3.94 – 4.84) vs. 1.97 (95% CI: 1.68 – 2.29) per 100.000 males and females, respectively (*p* < 0.001). No seasonal variation was observed.

**Conclusion:**

Over the past quarter century, the annual rates of newly diagnosed EoE patients raised dramatically and this increase has not reached a deceleration yet.

## Graphical abstract

Xxxxxx

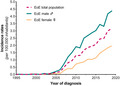

**Abbreviations**
CI: confidence intervals
EGD: esophagogastroduodenoscopy
EoE: eosinophilic esophagitis
eos: eosinophils
GERD: gastroesophageal reflux disease
GI: gastrointestinal
hpf: high‐power field
OR: odds ratio
PPI: proton‐pump inhibitor
Th2: T‐helper type 2

## 1 INTRODUCTION

Eosinophilic esophagitis (EoE) is a chronic immune‐mediated disease characterized by symptoms of esophageal dysfunction and infiltration of the mucosa with eosinophils.^1^, ^2^ Dysphagia and food impaction are the most typical complaints in adults, whereas gastroesophageal reflux disease (GERD)—symptoms and failure to thrive, feeding disorders, and abdominal pain—predominates in children.^1^, ^2^, ^3^ EoE is diagnosed per consensus guideline if i) there are symptoms of esophageal dysfunction and ii) ≥15 eosinophils (eos) per high‐power field (hpf) under routine light microscopy after hematoxylin and eosin staining are present in at least one esophageal biopsy.^4^ Over the past years, EoE has emerged from a case‐reportable illness in the early 1990s to a distinct clinicopathological entity.^5^, ^6^ Although clinicians are becoming more familiar with this relatively new disease, the expanded EoE frequency cannot be simply attributed to raised awareness alone and is outpacing any increase in diagnosis or detection.^7^, ^8^ The EoE epidemiology is rapidly evolving, and while genetic predisposition has been indicated, the increasing number of new EoE cases strongly suggests that (non‐)allergic environmental disparities may also be critical in disease manifestation.^3^, ^9^ A worldwide tendency of rising EoE incidences has been reported, though current estimates are inconsistent due to variety in study designs (eg, register‐based or insurance database vs. hospital‐based case series), heterogeneous reporting, and modified diagnostic criteria.^10^, ^11^, ^12^ Over successive years, the frequency of EoE in the Netherlands has also increased tremendously and it remains unclear whether this still continues to rise.^13^, ^14^ Hence, the Dutch register–based pathology database (PALGA) offers an unique opportunity to present an update of accurate EoE incidence rates with nationwide coverage.^11^, ^12^, ^15^ Therefore, we aimed to 1) assess the annual EoE incidence rates within the entire population in the Netherlands over the past 25 years and 2) to identify demographic trends (ie, gender, age, and date of diagnosis) over time.

## 2 METHODS

### 2.1 Data collection

This cross‐sectional study was conducted by using results from the nationwide network and registry from cyto‐ and histopathology in the Netherlands (PALGA).^15^ This archive contains data from all 46 pathology laboratories and has national coverage since 1991. Summarized histology reports are collected and encoded by pathologists based on the Systemized Nomenclature of Medicine (SNOMED) issued by the College of American Pathologists. By the end of 2017, more than 72 million pathology reports from over 12 million patients in the Netherlands have been archived in this database. All reports are encoded and comprise information on type of sample, macroscopic and microscopic features, and a final conclusion of the pathologist. Of note, our study was reviewed by the Medical Ethics Committee of our institution, the Amsterdam University Medical Centre (UMC) (W19_392 # 19.457).

### 2.2 Diagnostic criteria and case‐finding strategy

In this follow‐up study, a similar diagnostic framework for case identification was used as was previously published by our research group.^13^, ^14^ In addition to the previous search (1995–2015), the national database PALGA completed a comprehensive search to retrieve all pathology reports, matching the terms “esophagus” in combination with “eosinophilic inflammation”, “eosinophilic hyperplasia”, “eosinophilia”, “eosinophi”, or “allerg” between January 1, 2016, and December 31, 2019. All reports including primary carcinomas or describing eosinophilia in other regions of the gastrointestinal (GI) tract were excluded. After the first search, all duplicates were removed. All patients that were included in one of our previous search strategies between the years 1995 and 2015, without confirmation of diagnosis, were re‐reviewed and included if i) EoE was diagnosed based on a new pathology report and/or ii) EoE was suspected in retrospect based on previous reports and additional information with regard to the indication of performed esophagogastroduodenoscopy (EGD).^13^, ^14^ All cases were classified as EoE if 1) the diagnosis was confirmed by the pathologist and/or 2) the degree of esophageal eosinophilia in one biopsy sample (taken from ≥2 levels of the esophagus) was described as “markedly” (or words of comparable meaning), which was interpreted as similar to ≥15 eos/hpf by the reviewers (BDvR, MJW, WEdR, MEB). All reports describing “mild” (ie, moderate or words of similar meaning) esophageal eosinophilia without mentioning a peak eosinophil count of ≥15 eos/hpf were excluded. A manual review of all reports was performed by the first reviewer, and a second reviewer was asked in case of uncertainty to reach consensus. After the first manual review, additional information with regard to the indication of the performed EGD with biopsies was requested in case of uncertainty. A comprehensive evaluation of these reports including additional data was done by the reviewers, and cases were excluded if an EGD was performed due to suspicion of other potential causes of esophageal eosinophilia, such as drugs, parasitic infection, Cohn's disease, or GERD. Clinical information that was considered to further support the diagnosis of EoE included symptoms of dysphagia or food impaction, and typical endoscopic signs of EoE (eg, furrows, rings). Of note, in several cases the diagnosis of concomitant GERD was made if suggestive clinical signs were mentioned in the requested information with regard to the indication of the EGD performed. Endoscopic signs of reflux esophagitis or typical reflux‐related symptoms were interpreted as being suggestive for the diagnosis or GERD. After re‐review, all reports classified as EoE were included for final analysis. Furthermore, demographic data (gender, age, and date of diagnosis) and relevant histological features (spongiosis, micro‐abscesses, basal zone hyperplasia, and sub‐epithelial fibrosis) were derived from our database.

### 2.3 Endoscopy with esophageal biopsy sampling

To estimate the number of EGDs with esophageal biopsy sampling performed between 1995 and 2019, the PALGA database was queried. Search criteria were “esophagus” and “biopsy.” Of note, outcomes of this search yield an estimation of the total number of unique endoscopies with biopsies performed, considering that the search was not manually reviewed.

### 2.4 Statistical analysis

Statistical analysis was performed by using IBM SPSS Statistics (version 25.0) (SPSS, Chicago, USA). To calculate the annual incidence rates between 1995 and 2019 the total Dutch population was considered to be at risk for developing EoE. Crude incidence values were calculated by using the total number of newly diagnosed EoE patients and matched with Dutch population data (https://www.cbs.nl). Incidence rates were calculated for the entire population and stratified for gender and age. Descriptive statistics were used to assess demographic characteristics, presented as mean (±SD) for normally distributed continuous data and percentages (%) for categorical data. Groups were compared with chi‐square statistics and unpaired t test, as appropriate. A p‐value of 0.05 was considered to be statistically significant. Odds ratio (OR) and 95% confidence intervals (CI) were calculated by using MedCalc Software Ltd (Ostend, Belgium).

## 3 RESULTS

### 3.1 Case identification

**FIGURE 1:**
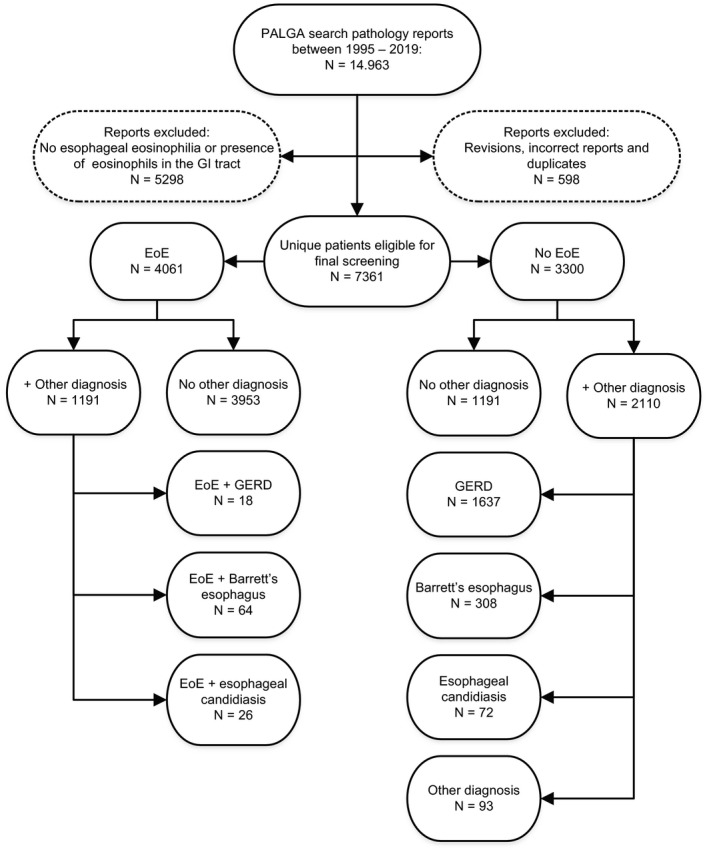
Flowchart of case identification. After the initial PALGA search, revisions, incorrect reports, and duplicates, as well as reports with absence of esophageal eosinophilia or presence of eosinophils in the gastrointestinal (GI) tract were excluded. A total of 7361 unique patients were eligible for review, of which 4061 cases were identified as eosinophilic esophagitis (EoE) in accordance with the conclusion of the pathologist

The database search between January 1, 1995, and December 31, 2019, included a total of 14.963 reports, of which 5298 reports were excluded due to non‐existing esophageal eosinophilia or the presence of eosinophils in the GI tract. In addition, another 598 reports, including revisions, incorrect reports, or duplicates (ie, double reports or previous EoE diagnosis between 1995 and 2016), were removed. In total, 7361 unique patients, who covered a total of 9068 reports with esophageal eosinophilia, were considered to be eligible for final inclusion. After the first review, 5076 unique reports were classified as “suspected for the diagnosis of EoE.” In total, 4061 unique patients were identified with a confirmed diagnosis of EoE following a second critical appraisal based on requested clinical information with regards to the indication of performed EGD (n = 3509). Of note, 31 patients being already included in our previous search (1995–2016) with no diagnosis of EoE were re‐reviewed, of which EoE was confirmed in 27 patients. In total, 3974 (98%) reports were diagnosed with EoE at the first EGD. Furthermore, 2110 patients were diagnosed with different disease entities other than EoE. A flowchart of case identification is presented in Figure 1.

### 3.2 Patient characteristics

**FIGURE 2:**
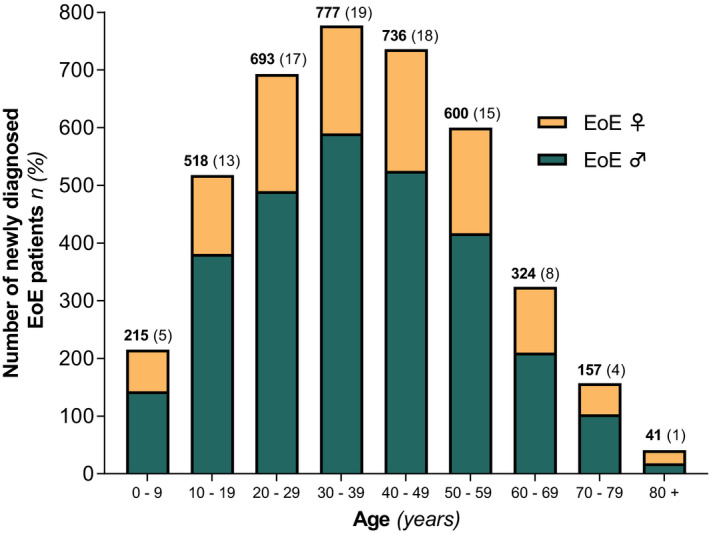
Distribution of age at diagnosis in male and female patients with eosinophilic esophagitis (EoE), presented in 10 years strata

A male predominance (71%) was confirmed in our cohort, with a mean age at diagnosis of 37.9 ± 18.4 years. In total, 639 (16%) children (<18 years) and 3422 (84%) adults were diagnosed. Of all identified adult EoE patients, 2419 (71%) were male and 1003 (29%) were female. The mean age at diagnosis in adults was 42.9 ± 15.4 years, with a significant higher age in females compared with males (44.5 ± 16.5 years vs. 42.2 ± 14.8 years; *p* < 0.001). In children, the mean age at diagnoses was 10.9 ± 5.3 years, of which no difference between males and females (11.1 ± 5.2 vs. 10.4 ± 5.5; *p* = 0.138) was observed. EoE was diagnosed at all ages (3:1 male‐to‐female ratio), with patients between the ages of 20 and 49 years being mostly affected. An overview of all newly identified EoE patients within the years of 1995 and 2019, stratified by gender and age, is presented in Figure 2.

**FIGURE 3:**
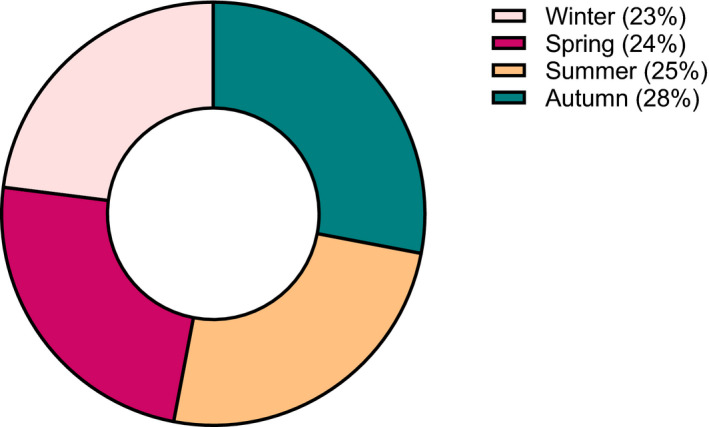
Seasonal distribution of newly diagnosed eosinophilic esophagitis (EoE) patients between 1995 and 2019 in the Netherlands

Furthermore, 108 patients were determined as EoE with a concomitant esophageal disease based on the conclusion of the pathologist. In total, 18 (17%) patients were identified with EoE and coexisting GERD, 64 (59%) patients with EoE and Barrett's esophagus, and 26 (24%) patients with EoE and esophageal candidiasis. In addition, no seasonal variations in the diagnosis of EoE were observed within the entire study timeframe (Figure 3).

### 3.3 Histological features

The degree of esophageal eosinophilia in all identified EoE patients was mentioned in 1608 (40%) unique reports, of which 1473 (36%) were classified as marked (ie, pronounced) and 135 (3%) as mild (ie, moderate). Of note, in all 135 reports describing “mild” esophageal eosinophilia, the diagnosis of EoE was confirmed and/or ≥15 eos/hpf were described by the pathologist. In only 588 (15%) reports, the esophageal eosinophilia was quantified as ≥15 eos/hpf within the entire study time frame. Between 1995 and 2009, quantification of the esophageal eosinophilia (ie ≥15 eos/hpf) was not stated in any of the reports. Between 2010–2014 and 2015–2019, the esophageal eosinophilia was described as ≥15 eos/hpf in 124 (3%) and 464 (12%), respectively, pathology reports. Additional histological features, such as; spongiosis, micro‐abscesses, basal zone hyperplasia, and sub‐epithelial fibrosis were described in less than 2% of all pathology reports.

### 3.4 Incidence rates of EoE

**FIGURE 4:**
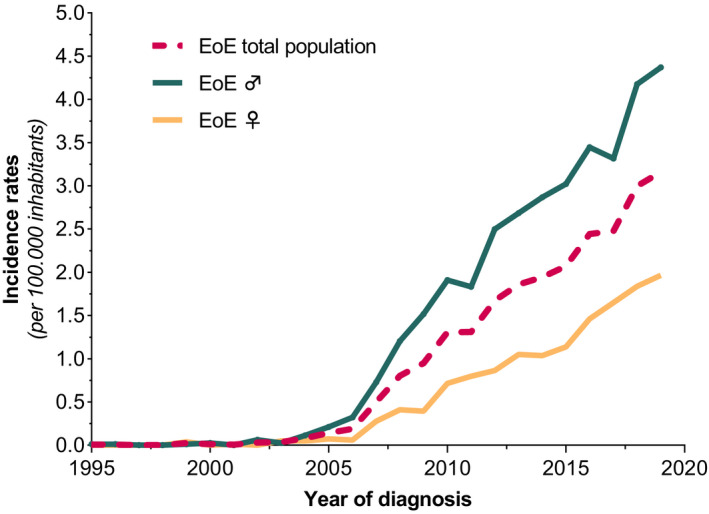
Incidence rates of eosinophilic esophagitis (EoE) in males and females between 1995 and 2019 in the Netherlands

**FIGURE 5:**
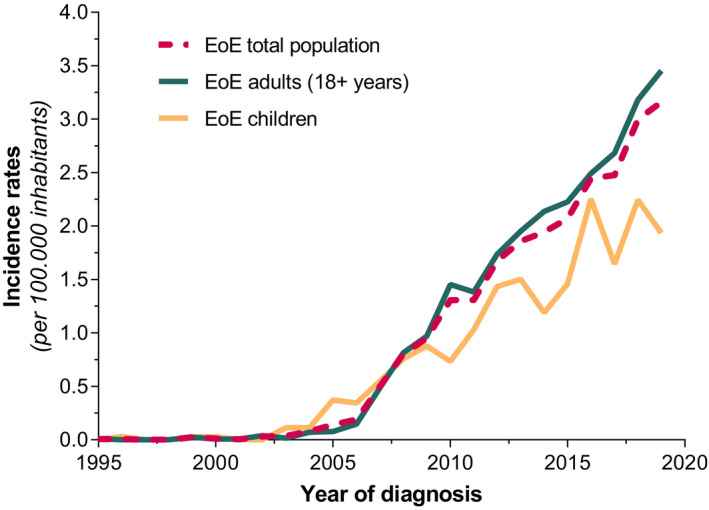
Incidence rates of eosinophilic esophagitis (EoE) in children and adults (18+ years) between 1995 and 2019 in the Netherlands

**FIGURE 6:**
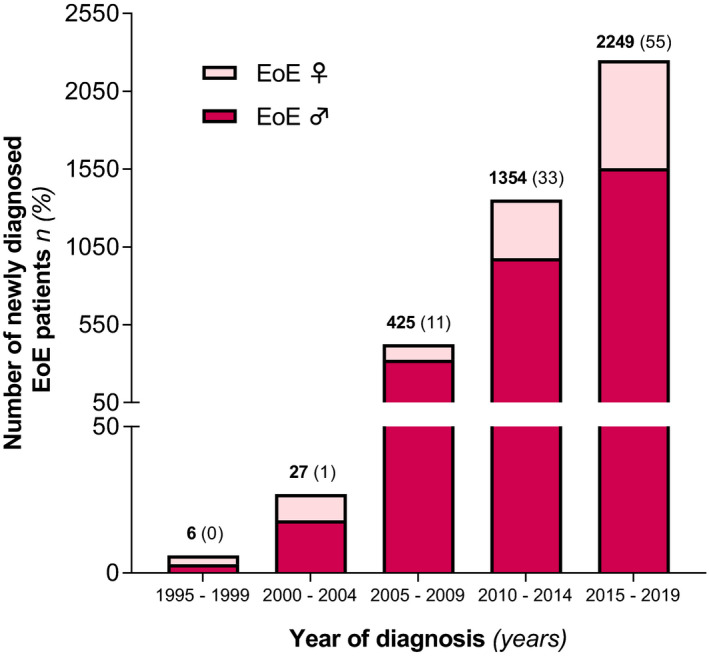
Distribution of year of diagnosis in male and female patients with eosinophilic esophagitis (EoE), presented in 5 years strata

The entire Dutch population comprised a total of 15.424.122 inhabitants in the year of 1995 and 17.282.163 inhabitants in the year of 2019 in accordance with the Central Bureau of Statistics (CBS). The average annual incidence of new cases per year during this time period is estimated to be 0.99 (95% CI: 0.84–1.15) per 100.000 inhabitants, based on the population of 16.390.837 citizens. Between the years of 1995 and 2004, the incidence rates increased slightly from 0.01 (95% CI: 0.0–0.04) new cases per 100.000 inhabitants in 1995 to 0.08 (95% CI: 0.04–0.14) new cases per 100.000 inhabitants in 2004. From then on, an impressive rise of yearly new EoE diagnosis was observed between 2005 and 2019, comprising rates of 0.14 (95% CI: 0.09–0.21) to 3.16 (95% CI: 2.90–3.44) new cases per 100.000 inhabitants. During the time period between 1995 and 2019, males were significantly more at risk for the presence of EoE compared with females (OR: 2.48 | 95% CI: 2.32–2.65; *p* < 0.001). Annual incidence rates of males and females between the years of 1995 and 2019 are presented in Figure 4. Over the past 25 years, adults were significantly more affected compared with children, with estimated average rates of 1.1 (95% CI: 0.89–1.3) and 0.7 (95% CI: 0.48–1.1), respectively, new cases per 100.000 inhabitants (OR: 1.46 | 95% CI: 1.34–1.59; *p* < 0.001). Trends of incidence rates in children and adults are demonstrated in Figure 5. The highest disease occurrence was observed in the final year of our analysis, with incidence rates in males and females of 4.37 (95% CI: 3.94–4.84) vs. 1.97 (95% CI: 1.68–2.29) per 100.000 inhabitants (*p* < 0.001). In 2019, EoE was mostly diagnosed in patients between the ages of 20 and 29, with significantly higher rates in males compared with females of 1.83 (95% CI: 1.45–2.28) vs. 0.90 (95% CI: 0.64–1.24 new cases per 100.000 inhabitants), respectively (*p* < 0.001). The majority of EoE patients (55%) were identified between the years of 2015 and 2019, with an estimated annual incidence over this time period of 2.63 (95% CI: 2.4 – 2.9) new EoE cases per 100.000 inhabitants. Distribution of year of diagnosis in 5 years strata for male and female patients is presented in Figure 6.

### 3.5 Endoscopy with esophageal biopsy sampling

**FIGURE 7:**
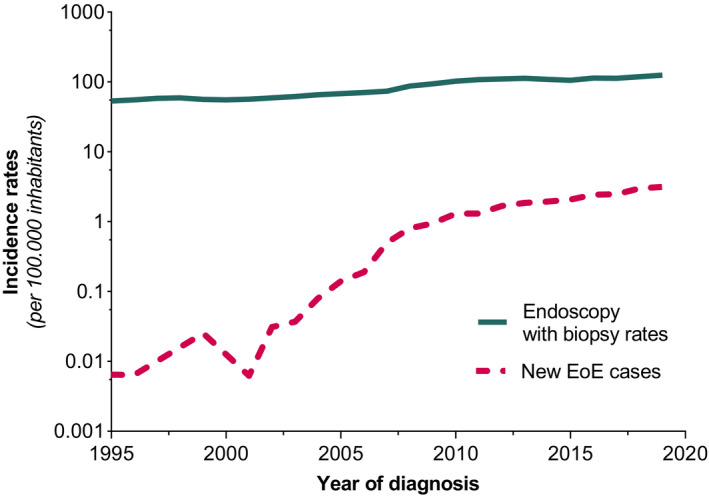
New EoE cases per 100.000 inhabitants per year and the estimated number of yearly performed endoscopies with esophageal biopsy sampling per 100.000 inhabitants, between the years of 1995 and 2019. Logarithmic y‐axes were used to visualize outcomes of different orders of magnitude in one graph

Within the study time frame, a 2.6‐fold increase in endoscopy with esophageal biopsy sampling was observed. The number of EGDs with biopsies performed per year increased from 8217 in 1995 per 100.000 inhabitants to 21.605 per 100.000 inhabitants in 2019 (Figure 7).

## 4 DISCUSSION

Observations on emerging incidence trends of EoE in the Netherlands are presented in this nationwide register‐based study. Over a quarter century, the incidence of EoE has expanded a 316‐fold and is still continuing to increase. A male predominance (3:1 ratio) was confirmed in this large cohort and patients between the ages of 29 and 49 years were most often affected. Within the entire study period, the EoE incidence was significantly higher in adults compared to children. These findings are consistent with previous literature, and the natural course of this chronic progressive disease.^7^, ^10^, ^16^, ^17^ Given the rise in EoE frequency and its non‐fatal nature, the prevalence in the Netherlands is estimated to be nearly 23.5 (95% CI: 22.8 – 24.2) EoE cases per 100.000 inhabitants in 2019 and has doubled again within the past 5 years. Although the estimated prevalence suggests that EoE is still a relatively rare disease by absolute numbers, the increasing and still ongoing frequency of new cases underscores the real magnitude of this emergent disease that is nearly approaching those of Crohn's disease in European countries.^18^, ^19^, ^20^ Moreover, the number of annual new EoE diagnosis increased from 0.01 new cases per 100.000 inhabitants in 1995 to 3.6 new cases per 100.000 inhabitants in 2019. These observations are similar to a register‐based study from Denmark reporting incidence rates of 0.13 to 2.6 new EoE cases per 100.000 inhabitants between the years of 1997 and 2012.^21^ Of note, other European population‐based EoE incidence rates were remarkably higher within this time frame compared with our observations.^22^, ^23^, ^24^ With regard to a meta‐analysis of Navarro *et al.*, the pooled annual incidence rates between the years 1989 and 2017 in North‐America and Europe were 7.1 and 2.7 cases per 100.000 inhabitants, respectively.^10^ However, our results are in the lower spectrum of previous reported findings, with an estimated annual average incidence rate of 0.99 cases per 100.000 inhabitants. Nevertheless, these discrepancies in EoE incidence rates among studies are more likely explained by heterogeneous case definition and study designs rather than geographic variation.

The ongoing rise of EoE incidences in the Netherlands is consistent with previous findings on EoE being an increasingly common disorder in developed countries.^10^ Several explanations have been suggested for the observed rise in prevalence in recent years. At first, increased detection bias following more widespread use of EGD with biopsies in general practice was considered a potential causative factor. However, we demonstrated only a 2.6‐fold expansion of endoscopies with esophageal biopsy sampling performed within the study window, whereas the incidence of EoE raised a 316‐fold. Moreover, multiple other studies have also confirmed that the increase in newly diagnosed patients far outpaces any expansion in EGD with biopsy.^8^, ^14^, ^23^ The overall dramatic rise of EoE frequency is paralleling other increasing Western diseases (eg, atopic morbidities and Crohn's disease), thereby suggesting a pivotal role for the environment in EoE pathogenesis.^25^, ^26^ Early childhood is known to be important for immune maturation; hence, developmental susceptibility might be influenced by early‐life experiences.^27^ It was therefore hypothesized that modern hygienic conditions may result in less exposure to microbes during infancy, subsequently causing a defect in immune tolerance and increased sensitivity to allergic diseases.^28^, ^29^ Moreover, early‐life events (eg, Cesarean section and antibiotic exposure) are considered to cause alterations of the composition and diversity of the microbiome, potentiating a T‐helper type 2 (Th2) immune‐mediated response in certain sensitive individuals.^30^, ^31^, ^32^ In addition, changes in environmental factors (eg, genetic modification, food additives, and water and/or air pollution) and a Western dietary pattern (ie, diet low in fibers and high in saturated fat) are also associated with microbial dysbiosis.^33^, ^34^, ^35^, ^36^ Moreover, the decline in frequency of *Helicobacter Pylori* infections and increasing prevalence of GERD in developed countries over the past several decades are both considered to contribute to the rapid rise of EoE.^37^, ^38^ Interestingly, also the emerging EoE frequency closely coincides with increased acid‐suppressant medication use, by that linking the rising use of proton‐pump inhibitors (PPIs) as a potentiating factor (ie, prevention of peptic digestion of food allergens and microbial dysbiosis) to the development of EoE.^39^, ^40^, ^41^, ^42^, ^43^, ^44^, ^45^ Taken all together, several mechanisms explaining the increase in EoE incidence have been suggested but none of these seems to offer a complete clarification. Although there is little evidence linking aeroallergen exposure to disease onset and flares, the exact role of allergic environmental factors in the pathogenesis remains unclear.^46^, ^47^ However, within the time frame of our study no seasonal variations in EoE diagnosis were observed.

Some methodological challenges were encountered during this study. No data were available on patients’ characteristics (eg, symptoms and medical history) due to the use of encoded PALGA pathology reports. Therefore, the majority of EoE diagnosis (52%) were exclusively based on histological information. Moreover, in only 588 (15%) reports the esophageal eosinophilia was quantified as ≥15 eos/hpf by the pathologist. These limitations of our diagnostic framework may have resulted in an underestimation of the observed EoE incidences. Nevertheless, 3509 (48%) reports were re‐reviewed by using additional information with regard to the indication of performed EGD in order to expand the reliability of our case‐finding strategy. Of note, a former medical chart review (ie, clinical, endoscopic, and histological data) was performed by our research group, by that affirming a clinicopathological EoE diagnosis in 721 (33%) randomly selected cases between 1995 and 2015.^48^ Moreover, all PALGA reports were consistently registered; hence, no further histological pathognomonic features were included in our diagnostic strategy. Despite these limitations, our study design has multiple strengths as well. At present, this is the largest population‐based study providing most recent data on EoE incidence rates within a 25‐year time period. In addition, the risk of selection bias was reduced by the consistent use of one similar diagnostic framework with nationwide coverage of histological data.^13^, ^14^ Regarding our diagnostic strategy, we consider these results to reflect a valid and consistent overview of EoE incidence rates over the past quarter century.

In conclusion, we present observations on escalating EoE incidences over a considerable time frame of 25 years in the Netherlands. From these results, it is clear that EoE incidence has not stabilized yet and continues to rise. These findings underscore the need to further investigate the mechanisms underlying its pathogenesis and which dynamic environmental components could lead to such an expansion of EoE cases.

## CONFLICT OF INTEREST

WEdR, MEB, MJW, BDvR, AHB, and JV have no conflicts of interest. AJB has received research funding from Nutricia, SST, Norgine, and Bayer; and speaker and/or consulting fees from Laborie, EsoCap, Medtronic, DrFalk, Calypso, Regeneron, Celgene, Norgine, and AstraZeneca and owns stocks SST.

## **AUTHOR** **CONTRIBUTION**

WEdR is the guarantor of the article. WEdR, MJW, BDvR, JV, and AJB assisted in writing. WEdR, MJW, BDvR, and AJB conceived and designed the study. WEdR, MEB, MJW, BDvR, AHB, and AJB involved in generation, collection, assembly, analysis, and/or interpretation of data. WEdR prepared draft of the article. WEdR, MEB, MJW, BDvR, JV, AHB, and AJB approved the final version of the manuscript.
